# Caffeine-Induced Upregulation of *pas-1* and *pas-3* Enhances Intestinal Integrity by Reducing Vitellogenin in Aged *Caenorhabditis elegans* Model

**DOI:** 10.3390/nu16244298

**Published:** 2024-12-12

**Authors:** Mijin Lee, Jea Lee, Dongyeon Kim, Hyemin Min, Yhong-Hee Shim

**Affiliations:** 1 Department of Bioscience and Biotechnology, Konkuk University, Seoul 05029, Republic of Korea; miranda12@konkuk.ac.kr (M.L.); hyeminmin@kaist.ac.kr (H.M.); 2 Department of Systems Biotechnology, Konkuk University, Seoul 05029, Republic of Korea; jealee@konkuk.ac.kr (J.L.); banggae777@konkuk.ac.kr (D.K.)

**Keywords:** caffeine, *Caenorhabditis elegans*, *pas-1*, *pas-3*, intestinal health, yolk protein

## Abstract

**Background:** Intestinal aging is characterized by declining protein homeostasis via reduced proteasome activity, which are hallmarks of age-related diseases. Our previous study showed that caffeine intake improved intestinal integrity with age by reducing vitellogenin (VIT, yolk protein) in *C. elegans*. In this study, we investigated the regulatory mechanisms by which caffeine intake improves intestinal integrity and reduces vitellogenin (VIT) production in aged *Caenorhabditis elegans*. **Methods:** We performed RNA-seq analysis, and qRT-PCR to validate and confirm the RNA-seq results. Transgenic worms with VIT-2::GFP and VIT-6::GFP were used for measuring VIT production. dsRNAi was conducted to elucidate the roles of *pas-1* and *pas-3* genes. **Results:** *pas-1* and *pas-3*, a *C. elegans* ortholog of human *PASM4*, was upregulated by caffeine intake. They reduced VIT production by repressing *unc-62*, a transcriptional activator of *vit* expression. Interestingly, *vit-2* was required for *pas-1* and *pas-3* expression, and RNAi of *pas-1* and *pas-3* promoted intestinal atrophy and colonization, suggesting a balancing mechanism for VIT levels in intestinal health. Additionally, lifespan was extended by caffeine intake (2 ± 0.05 days), however, this effect was not observed by *pas-1* but not *pas-3* RNAi, suggesting that the mode of action for an anti-aging effect of caffeine through *pas-1* and *pas-3* is distinctive. The lifespan extended by *pas-1* was mediated by SKN-1 activation. **Conclusions:** Caffeine intake enhances intestinal health through proteasome activity and extends lifespan in aged *C. elegans* by upregulating *pas-1* and *pas-3*. These findings suggest that caffeine consumption mitigates age-related proteasome impairment and maintains intestinal integrity during aging.

## 1. Introduction

Diet is intrinsically connected to intestinal health, and subsequently linked to a healthy life. Among dietary components, caffeine is a component consumed world-wide that causes multiple physiological effects. It is rapidly absorbed in the stomach and small intestine, enhancing the activity of detoxifying enzymes that alter metabolism [1]. Caffeine presents in various dietary sources including chocolate, coffee, green tea, and energy drinks [2]. The beneficial or harmful effects of caffeine depend on its concentration, duration of consumption, and age across different organisms [3,4,5,6,7,8,9,10,11,12]. Many studies in various species have shown that caffeine has neuroprotective effects in Alzheimer’s and Parkinson’s patients by enhancing memory function while potentially increasing anxiety and negatively affecting sleep [3,4,5,6,7,8,9,10,11,12]. Additionally, it contributes to intestinal health by improving colonic motor function and altering intestinal microbial communities [13,14,15,16]. The alteration in the intestinal microbiome due to dietary intake is known to be linked to several diseases such as inflammatory bowel disease, obesity, and diabetes [17]. These chronic diseases influence lifespan by various biological and genetic pathways [18]. Dietary factors are further known to modulate adult disorders and aging beyond genetic predispositions [19,20,21]. Therefore, elucidating the regulatory mechanisms between dietary factors and intestinal health is necessary. For this purpose, *Caenorhabditis elegans*, a simple animal model, provides several advantages for studying intestinal health: its transparent body allows direct observation of the intestine, its short lifespan enables rapid aging studies, and importantly, it shares remarkable similarities with mammals in intestinal aging phenotype [22,23]. For instance, both *C. elegans* and mammals exhibit comparable age-related intestinal deterioration, characterized by compromised epithelial permeability, reduced and shorter microvilli, and accumulation of undigested dietary components [22,23].

In *C. elegans*, vitellogenin (VIT, yolk protein) supports embryonic development and exhibits similarities to low-density lipoproteins (LDL) in humans based on sequence homology [24,25,26]. VIT expression persists during the aged adult stage in *C. elegans*, causing aging-related intestinal atrophy through continuous production even after post-reproduction [24,27,28]. In humans, LDL levels are similarly implicated in age-related diseases, reflecting the detrimental effects of VIT in the intestine of an aged *C. elegans* [25,28,29]. These intestinal dysfunctions are key aging markers that influence organismal lifespan [23,30].

We have reported diverse effects of caffeine on biological processes in the *C. elegans* model [5,8,10,11]. In particular, we found protective effects of caffeine intake on intestinal aging in aged *C. elegans* [10]. A previous study demonstrated that caffeine intake decreases VIT production, which enhances intestinal health and extends lifespan. Additionally, it shows antioxidant effects on intestinal health by enhancing mitochondrial function, contributing to the extension of lifespan through SKN-1 activity [10]. However, it remains largely unknown how caffeine intake enhances intestinal health and lifespan associated with vitellogenesis in aged *C. elegans*. Therefore, in this study, we investigated the regulatory mechanisms induced by caffeine intake in intestinal health and lifespan extension through VIT production. Our findings indicate that caffeine intake enhances intestinal health and extends lifespan by upregulating the proteasome α-subunit *pas-1* and *pas-3* in aged *C. elegans*.

## 2. Materials and Methods

### 2.1. Caenorhabditis elegans Strains and Caffeine Treatment

**Figure 1:**
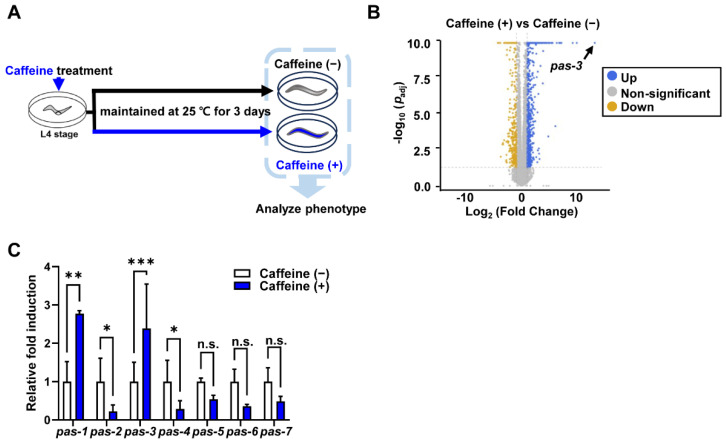
Caffeine intake increased expressions of *pas-1* and *pas-3* in aged *C. elegans*. (**A**) Experimental scheme for caffeine intake in aged *C. elegans*. (**B**) The volcano plot shows differentially expressed genes in caffeine-intake worms compared to caffeine-free worms (A total of 6 samples were used, with 300 worms per condition). Genes with increased expression (fold-change value ≥ 2 and *p*-value < 0.05) are shown in blue, and genes with decreased expression (fold-change value ≤ 2 and *p*-value < 0.05) in orange. (**C**) Relative *pas* family gene mRNA levels in the worms with or without caffeine intake were quantified by qRT-PCR. The graph shows the average fold-changes of each *pas* gene mRNA normalized with the level of *act-1* mRNA. Error bars indicate standard deviation. *n* = 300 per each condition. n.s., not significant; *, *p* < 0.05; **, *p* < 0.01; ***, *p* < 0.001 (two-way ANOVA with Tukey’s post hoc test).

Strains were grown at 15 °C on nematode growth medium agar plates seeded with *Escherichia coli* OP50 as previously described [31]. The following strains were used: N2 (wild type, Bristol), DH1033: *bIs1 (vit-2::GFP+rol-6(su10060))* X, BC12843: *dpy-5(e907)* I; *sIs11286(rCesK07H8.6(vit-6)::GFP+pCeh361)*, RB2365: *vit-2(ok3211)* X, ERT60: *jyIs13 [act-5p::GFP::ACT-5+rol-6(su1006)]* II, LD1: *Idls7[skn-1b/c::GFP+rol-6(su1006)]*. In our previous studies, we decided to use 10 mM caffeine to observe its effects on intestinal aging and vitellogenesis because caffeine at concentrations below 10 mM had little effect on worms, while concentrations above 10 mM were toxic [5,8,10]. The synchronized L4 staged worms were treated with 0 mM or 10 mM caffeine (Sigma-Aldrich, St. Louis, MO, USA) at 25 °C for 72 h, as previously described in [10]. The experimental scheme is shown in Figure 1A.

### 2.2. RNA Sequencing and Data Visualization

Total RNA was extracted from *C. elegans* fed with 0 or 10 mM caffeine at 25 °C for 72 h and sequenced by Macrogen Inc. (Seoul, Republic of Korea) using the Illumina NovaSeq platform with paired-end reads. Each sample consisted of 300 worms, with three samples for each condition, a total of six samples for analysis. Initial data preprocessing involved employing Trimmomatic version 0.38 to eliminate adapter contaminations and remove low-quality base calls. Subsequent read alignment was performed against the *C. elegans* reference genome (WBcel235) utilizing HISAT version 2.1.0, leveraging established alignment methodologies. Genome sequence and annotation data were retrieved from the NCBI Genome assembly and RefSeq databases. Post-alignment, SAMtools version 1.9 was utilized for SAM file sorting and indexing. Transcript reconstruction and quantification were accomplished through StringTie version 2.1.3b, generating gene- and transcript-level measurements including raw read counts, FPKM, and TPM. Differentially expressed genes were visualized with a volcano plot, showing log_2_ fold changes and −log_10_ adjusted *p*-values from DESeq2 analysis. Its significance was determined through DESeq2’s negative binomial Wald Test, extracting fold change and *p*-value metrics. Multiple testing corrections utilized the Benjamini–Hochberg procedure to manage the false discovery rate. Statistical significance for functional annotations was calculated using a one-sided hypergeometric test with Benjamini–Hochberg correction.

### 2.3. RNA Extraction and Quantitative Reverse Transcriptase-PCR (qRT-PCR)

Total RNA was isolated using TRIzol reagent following the manufacturer’s protocol (Takara Bio Inc., Shiga, Japan). To synthesize the cDNA, the extracted RNA was reverse-transcribed using oligo-dT primer and M-MLV reverse transcriptase (Invitrogen, Waltham, MA, USA). qRT-PCR was performed using the Applied Biosystems 7500 Real-Time PCR System. Each 10 μL reaction contained 50–100 ng of cDNA template, 10 pM of each primer, and Power SYBR Green PCR Master Mix (Applied Biosystems, Waltham, MA, USA). The thermocycling conditions were: 50 °C for 2 min, 95 °C for 10 min, followed by 40 cycles of 95 °C for 15 sec and 60 °C for 1 min. A dissociation curve analysis was performed to confirm amplification specificity, with a single peak observed for all primers. *act-1* was selected as the reference gene based on its established stability across different experimental conditions in *C. elegans* [32]. The used primers are listed in Supplementary Table S1.

### 2.4. RNA Interference (RNAi)

For the soaking RNAi treatments of *pas-1* and *pas-3*, each of the double-stranded RNA was transcribed *in vitro* from respective cDNA templates, which were PCR-amplified with primers as described previously [33]. The synchronized L4 staged worms were soaked for 24 h in each RNAi solution and then transferred to 0 mM or 10 mM caffeine plates. RNAi efficiency in this analysis was confirmed by observing increased embryonic lethality, a prominent phenotype of *pas-1* and *pas-3* RNAi (Supplementary Figure S1, [34]).

### 2.5. Intestinal Integrity Assays

For intestinal integrity studies, we used three kinds of methods, including intestinal atrophy, bacterial colonization, and intestinal localization, which are critical for evaluating intestinal health because they directly reflect the structural and functional state of the intestine [10,27]. Intestinal atrophy indicates the degeneration of intestinal tissues, which can impair nutrient absorption and overall gut function [27]. Bacterial colonization is essential to monitor because an imbalance in intestinal microbiota can lead to dysbiosis [35]. For intestinal atrophy analysis, the intestinal width posterior to the vulva was measured, the lumenal width was subtracted, and the result was divided by the body width, as previously described [10,27]. Expression of ACT-5::GFP which is a marker for actin cytoskeleton integrity was measured under a fluorescence microscope (Zeiss Axioscope, Oberkochen, Germany) for intestinal localization. For intestinal colonization analysis, worms were fed with OP50::GFP, a fluorescent bacteria, on NGM plates containing 0 or 10 mM caffeine and observed under a microscope (Zeiss Axioscope, Oberkochen, Germany).

### 2.6. Motility and Life Span Assays

For motility analysis, each worm was soaked in 10 µL of M9 buffer on NGM plates, and body bends were counted every 20 s. A body bend was defined as a complete cycle of terminal bulb motion, as described in [10]. For lifespan analysis, worms were observed daily until all worms were dead, and the number of dead and live animals was counted.

### 2.7. Statistical Analysis

All experiments were performed in triplicate. The graph and statistical analyses were performed using Prism GraphPad 10 software version 10. 4. 0 (621), (https://www.graphpad.com/, accessed on 23 October 2021). The *p*-values were calculated using one- or two-way ANOVA for statistical evaluation and the details about the statistical methods for the data are provided in the figure legends. For statistical analysis of percentage data, data were analyzed using the chi-square test, with *p* < 0.05 considered statistically significant. Significance was considered at *p* < 0.05 (*), *p* < 0.01 (**), *p* < 0.001 (***).

## 3. Results

### 3.1. Caffeine Intake Significantly Increases Expression of Proteasome α-Subunit Genes, pas-1 and pas-3 in aged C. elegans

To elucidate the molecular mechanism underlying reduction in vitellogenesis (yolk protein production) by caffeine intake in aged *C. elegans*, we performed RNA sequencing (RNA-seq) using caffeine-fed or non-fed control groups of *C. elegans* grown at 25 °C for 3 days (Figure 1A). The RNA-seq analysis revealed a significant increase in the expression of *pas-3*, an orthologue of the human type-4 α-subunit gene [36,37] with an upregulation of approximately 10,000-fold compared to the control by caffeine intake (Figure 1B; Supplementary Table S2). This result suggests a potential key role for *pas-3* in the observed anti-aging effects of caffeine intake which have been previously reported [10]. Given that there are seven family members of *pas* genes in 20S α-type proteasome subunits in *C. elegans* [36,37], we determined whether the other members of *pas* genes were also upregulated by caffeine intake by validating the expression levels of *pas* family genes using qRT-PCR. Interestingly, the mRNA levels of *pas-1* and *pas-3* were significantly increased whereas those of the rest of the *pas* genes, *pas-2*, *pas-4*, *pas-5*, *pas-6*, and *pas-7*, decreased (Figure 1C). These findings suggest the differential regulation of each *pas* gene in response to caffeine intake during aging in *C. elegans*.

### 3.2. Caffeine Intake Reduces Vitellogenin Production by Modulating pas-1 and pas-3 During Aging in C. elegans

**Figure 2:**
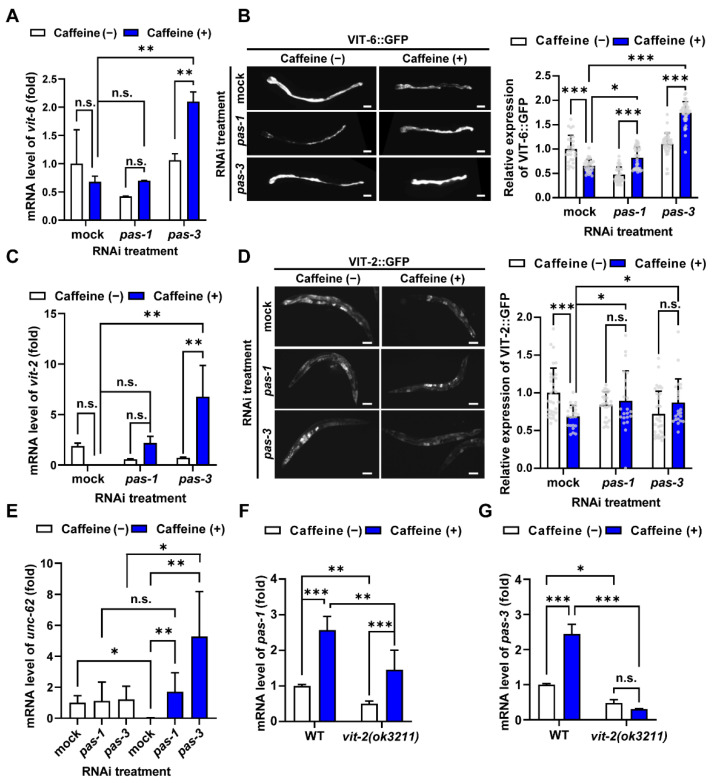
Caffeine intake reduced vitellogenin by mediating *pas-1* and *pas-3* in aged *C. elegans*. (**A**) Relative mRNA levels of *vit-6* in the worms with or without caffeine intake following *pas-1* or *pas-3* RNAi were quantified by qRT-PCR. (**B**) The representative images and graph show the expression of VIT-6::GFP with or without caffeine following *pas-1* or *pas-3* RNAi (*n* = 28, 37, 41, 41, 44, 41). Error bars represent standard deviation (s.d.). *, *p* < 0.05; *** *p* < 0.001 (two-way ANOVA with Tukey’s post hoc test). Grey dots represent individual data points. Scale bars, 10 µm. (**C**) Relative mRNA levels of *vit-2* in the worms with or without caffeine intake following *pas-1* or *pas-3* RNAi were quantified by qRT-PCR. (**D**) The representative images and graph show the expression of VIT-2::GFP with or without caffeine following *pas-1* or *pas-3* RNAi (*n* = 33, 24, 27, 25, 35, 32). Error bars represent s.d. n.s., not significant; *, *p* < 0.05; *** *p* < 0.001 (two-way ANOVA with Tukey’s post hoc test). Grey dots represent individual data points. Scale bars, 10 µm. (**E**) Relative mRNA levels *unc-62* in the worms with or without caffeine intake following *pas-1* or *pas-3* RNAi were quantified by qRT-PCR. (**F**,**G**) Relative mRNA levels *pas-1* (**F**) or *pas-3* (**G**) in wild-type N2 and *vit-2(ok3211)* mutant with or without caffeine intake. * *p* < 0.05; ** *p* < 0.01; *** *p* < 0.001 (two-way ANOVA with Tukey’s post hoc test). All qRT-PCR graphs show the average fold-changes of mRNA normalized to the level of *act-1* mRNA. *n* = 300 per each condition. Error bars indicate s.d., n.s., not significant; * *p* < 0.05; ** *p* < 0.01; *** *p* < 0.001 (two-way ANOVA with Tukey’s post hoc test).

In many species, proteasome activity decreases with age, leading to protein accumulation that can induce age-related diseases [38,39]. Specifically, continuous VIT production and accumulation in the intestine during *C. elegans* aging accelerates the aging process [24,27]. We previously found that caffeine intake reduced VIT production and slowed aging [10]. Given these findings, we hypothesized that caffeine intake decreases VIT production by increasing the expression of *pas-1* and *pas-3* during *C. elegans* aging. To test this possibility, we measured the mRNA levels of both *vit-6* and *vit-2,* as well as the expression levels of VIT-6::GFP and VIT-2::GFP in transgenic worms after *pas-1* or *pas-3* RNAi treatment in caffeine-fed and non-fed worms. The synchronized L4-staged worms were exposed to *pas-1* and *pas-3* RNAi treatments and were examined for VIT-6 and VIT-2 expression in an aged adult *C. elegans* (Figure 2A–D). RNAi efficiency in this analysis was confirmed by observing increased embryonic lethality, a prominent phenotype of *pas-1* and *pas-3* RNAi (Supplementary Figure S1, [34]). Caffeine intake did not significantly decrease the mRNA levels of *vit-6* and *vit-2* in mock and *pas-1* RNAi-treated worms although their average expression levels seemed to increase. Contrarily, the mRNA levels of *vit-6* and *vit-2* significantly increased in *pas-3* RNAi-treated worms (Figure 2A,C). These results suggest that *pas-3* transcriptionally represses the expression of *vit-6* and *vit-2*, increasing the mRNA expression under caffeine-fed conditions. Furthermore, caffeine intake significantly reduced the accumulation of VIT-6::GFP and VIT-2::GFP in mock-treated worms (Figure 2B,D). However, *pas-1* or *pas-3* RNAi-treated worms exhibited an increase in VIT-6::GFP and VIT-2::GFP expression in response to caffeine compared to mock-treated worms (Figure 2B,D), indicating that *pas-1* and *pas-3* reduced VIT accumulation in response to caffeine intake. These results indicate that caffeine intake decreases VIT expression by upregulating *pas-1* and *pas-3*. In our previous studies, we found that caffeine reduces VIT production by decreasing the expression of a transcription activator, *unc-62* [10,40]. To better understand this mechanism, we investigated whether *pas-1* and *pas-3* genes might regulate VIT through *unc-62* in response to caffeine. We measured *unc-62* mRNA levels under different conditions. In control worms (mock RNAi), caffeine intake decreased *unc-62* mRNA levels as expected (Figure 2E). However, either *pas-1* or *pas-3* RNAi increased *unc-62* mRNA levels in caffeine-fed worms (Figure 2E). This suggests that both *pas-1* and *pas-3* are necessary to repress *unc-62* expression under caffeine intake conditions. Interestingly, we observed a difference between *pas-1* and *pas-3* effects. In *pas-1* RNAi-treated worms, caffeine intake did not alter *unc-62* mRNA levels (Figure 2E). In contrast, *pas-3* RNAi in caffeine-fed worms led to a significant increase in *unc-62* mRNA compared to worms without caffeine (Figure 2E). This difference suggests that *pas-3* plays a more crucial role than *pas-1* in reducing VIT production, primarily by controlling *unc-62* expression under caffeine intake conditions. We further examined whether VIT mutually affects *pas* gene expression and *vice versa* by using *vit-2(ok3211)* mutants. Caffeine intake significantly decreased the mRNA levels of *pas-1* and *pas-3* in *vit-2(ok3211)* mutants compared to those in wild-type N2 worms under the same caffeine intake conditions (Figure 2F,G). It suggests that VIT enhances the induction of *pas-1* and *pas-3* gene expression by caffeine intake. Interestingly, in *vit-2(ok3211)* mutants, under caffeine intake, a significant increase in *pas-1* and almost abolished expression of *pas-3* were observed (Figure 2F,G), indicating that VIT expression is essential for the induction of *pas-1* and mainly *pas-3* expression in response to caffeine intake. This result suggests that *pas-1* and *pas-3* expressions are significantly induced in the presence of VIT, which represses *unc-62* expression and decreases *vit* gene expression under caffeine intake. By these processes, the balanced VIT production by *pas-1* and *pas-3* in caffeine-fed conditions appears to reveal an anti-aging effect.

### 3.3. Caffeine Intake Improves Intestinal Integrity via pas-1 and pas-3 in Aged Adults

**Figure 3:**
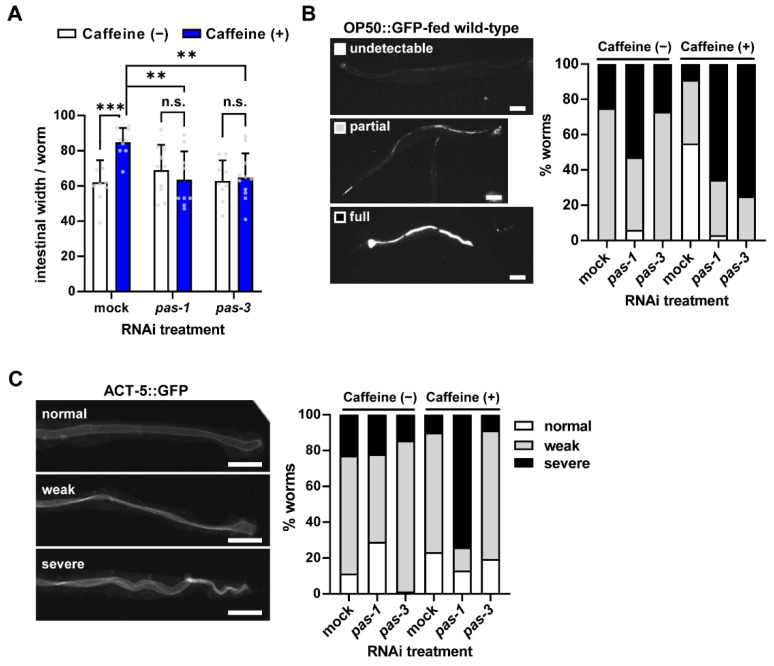
The improved intestinal health by caffeine intake in aged *C. elegans* was suppressed by either *pas-1* or *pas-3* RNAi knockdown. (**A**) The intestinal atrophy was measured in wild-type N2 with or without caffeine following *pas-1* or *pas-3* RNAi (*n* = 9, 9, 10, 9, 9, 11). n.s., not significant; ** *p* < 0.01; *** *p* < 0.001 (two-way ANOVA with Tukey’s post hoc test). Grey dots represent individual data points. (**B**) The bacterial colonization was shown in wild-type N2 with or without caffeine following *pas-1* or *pas-3* RNAi (*n* = 32, 32, 51, 39, 31) by feeding with *E. coli* OP50::GFP, a fluorescent bacteria. The bacterial colonization was classified into three categories: undetectable (white), partial (gray), and full (black). Scale bars, 50 µm. (**C**) Expression of ACT-5::GFP was treated with RNAi of *pas-1* or *pas-3* with or without caffeine intake (*n* = 17, 13, 21, 16, 25, 20). The type of actin mislocalization of the intestine was classified into three categories: normal (white), weak (gray), and severe (black). Scale bars, 50 µm.

We have previously reported that caffeine intake maintains intestinal integrity in aging worms by mitigating intestinal atrophy, reducing intestinal colonization, and maintaining Actin 5 (ACT-5) localization [10]. All of these intestinal improvements were possibly due to the reduction in VIT production [10]. Given our findings showing caffeine-intake-induced *pas-1* and *pas-3* reduced VIT production during aging, we investigated whether caffeine-induced *pas-1* and *pas-3* also contribute to maintaining intestinal integrity in aging worms. With aging, the suppression of severe intestinal atrophy by caffeine intake was significantly abolished with either *pas-1* or *pas-3* RNAi (Figure 3A), suggesting that the beneficial effects of caffeine intake on intestinal atrophy are mediated by *pas-1* and *pas-3.*

Consistent with previous reports, we observed that caffeine intake reduced intestinal colonization of GFP-expressing *E. coli* with age (Figure 3B). However, either *pas-1* or *pas-3* RNAi promoted intestinal colonization under caffeine intake conditions (Figure 3B). This finding was confirmed by the observation of intestinal localization using an ACT-5::GFP transgene, indicating the formation of intestinal microvilli (Figure 3C) [41]. *pas-1* RNAi-treated worms exhibited severe intestinal mislocalization but not *pas-3* RNAi-treated worms upon caffeine intake (Figure 3C), suggesting that *pas-1* plays a critical role in maintaining intestinal ACT-5-dependent localization. Taken together, both *pas-1* and *pas-3* are involved in maintaining intestinal integrity by caffeine intake.

### 3.4. pas-1 Extends SKN-1-Mediated Lifespan During Aging in Caffeine-Fed Aged C. elegans

**Figure 4:**
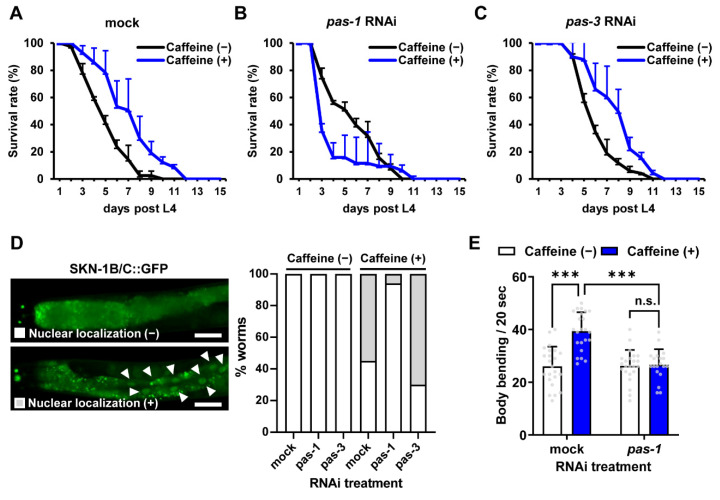
The skinhead 1 (SKN-1)-mediated lifespan extension by caffeine intake was suppressed by *pas-1* RNAi knockdown in aged *C. elegans.* (**A**–**C**) Comparison of the lifespan between the caffeine-free, caffeine (−) and caffeine-intake, caffeine (+), worms with mock (**A**), *pas-1* (**B**), or *pas-3* (**C**) RNAi (*n* = 45 per each condition). Worms were incubated at the L4 stage until dead at 25 °C. (**D**) Expression of SKN-1::GFP was measured in worms treated with RNAi of *pas-1* or *pas-3* with or without caffeine intake (*n* ≥ 15 per each condition). White bars represent SKN-1 remained in the cytoplasm, while grey bars indicate SKN-1 nuclear translocation. The white arrowheads indicate the translocation of SKN-1::GFP to the nucleus. Scale bars, 20 µm. (**E**) Comparison of body bending between caffeine-free (−) and caffeine intake (+) in RNAi of *pas-1* or *pas-3*-treated worms (*n* = 15 per each condition). Error bars represent standard deviation. n.s., not significant; *** *p* < 0.001 (two-way ANOVA with Tukey’s post hoc test). Grey dots represent individual data points.

Based on the previous findings that caffeine intake maintained intestinal integrity and extended lifespan by mediating SKN-1 activation [10], we examined whether *pas-1* and *pas-3* are involved in this process. Consistent with the previous report, caffeine intake extended the lifespan (2 ± 0.05 days, 34.2% increase) (Figure 4A, [10]). However, in *pas-1* RNAi conditions, caffeine intake reduced the mean lifespan from 5.67 ± 2.21 to 4.61 ± 2.08 days (18.7% decrease) (Figure 4B). In contrast, *pas-3* RNAi increased the mean lifespan from 4.81 ± 1.73 to 5.85 ± 2.07 days (21.6% increase) (Figure 4C). These results suggest that *pas-1* is required for the lifespan extension induced by caffeine intake.

Previously, it was reported that SKN-1 was activated in aged adults by caffeine intake and extended lifespan [10]. Therefore, we assessed whether *pas-1* and *pas-3* are involved in the extension of lifespan through SKN-1 activity in caffeine-fed worms. Notably, caffeine intake-induced SKN-1 activation was suppressed by *pas-1* but not *pas-3* RNAi (Figure 4D), indicating that *pas-1* is involved in the SKN-1-mediated lifespan extension by caffeine intake. We also confirmed that caffeine intake in *pas-1* RNAi-treated worms decreased motility, as assessed by a reduction in body bending counts (Figure 4E). Taken together, these findings suggest that *pas-1* and *pas-3* exert distinct roles for the anti-aging effects of caffeine during aging of *C. elegans* and *pas-1* is primarily associated with the extended lifespan.

## 4. Discussion

In this study, we identified that expression of *pas-3*, a *C. elegans* ortholog of human *PASM4*, was highly increased in caffeine-fed worms by RNA-seq analysis and qRT-PCR. Among seven family members of the *pas* gene, expressions of *pas-1* and *pas-3* were significantly increased by caffeine intake. PAS proteins form 20S proteasome α-subunit. The 20S proteasome subunits are essential for proteasome structure and function [37]. The α-type subunits are located in the outer rings of the core proteasome, and they bind to cap structures or regulatory particles that recognize polyubiquitin tags on protein substrates, initiating degradation [37]. Our findings suggest a link between caffeine intake and proteasome activity. However, the molecular mechanisms underlying caffeine-mediated upregulation of *pas-3* in *C. elegans* remain to be elucidated. In *C. elegans*, *pas-3* mutant with N-terminal truncation revealed over-expression with a hyperactive proteasome activity. Interestingly, the *pas-3* mutant exhibited phenotypes similar to those observed in caffeine-fed worms, including extended lifespan, reduced offspring fecundity, increased stress resistance, and shorter body length [10,42]. These findings raise the possibility that increased expression of *pas-3* by caffeine intake enhances proteasome activity which is responsible for various physiological changes in caffeine-fed worms. In humans, diabetic patients, PSMA4 was identified *in silico* analysis as a major target protein of coffee consumption [43]. Notably, our findings in *C. elegans* demonstrate that caffeine-induced upregulation of *pas-3* reduces vitellogenin (VIT) production, resulting in protection against intestinal aging. In humans, elevated levels of lipoprotein have been associated with metabolic disorders, including diabetes, potentially due to their roles in lipid transport and accumulation [44]. This evolutionary conservation of the *PSMA4*/*pas-3* pathway by caffeine intake between humans and *C. elegans* provides the potential therapeutic implications of caffeine in age-related metabolic disorders [45]. The regulatory mechanisms are unclear; however, it may involve direct or indirect molecular interactions between caffeine and specific structural motifs of *pas-3* or potentially through undefined factors.

Interestingly, differential mRNA levels of *pas* genes were induced by caffeine intake: *pas-1* and *pas-3* were increased, but not the other *pas* genes, suggesting distinctive responses to caffeine consumption among *pas* genes. In addition to *pas* genes, it would be necessary to examine other factors in proteasome subunits to see if they also respond to caffeine. It has been reported that the 20S proteasome α-subunit is ubiquitously expressed in the cytoplasm and nucleus in *C. elegans* [46]. However, their expressions vary across different tissues in response to stress due to compensatory regulation [46,47]. For instance, RNAi of either *pas-5* or *pas-6* increased proteasome expression in intestinal and body-wall muscle cells, while it decreased in oocytes, germ cells, and embryos [46]. These findings suggest a complex compensatory mechanism where different tissues modulate *pas* gene expression to maintain proteasome function under varying conditions. In our study, *pas-1* and *pas-3* appeared to respond to caffeine intake to compensate for VIT amount for intestinal health in aged adults. We found that expressions of *pas-1* and *pas-3* were decreased in the *vit-2* loss-of-function mutant, suggesting that their expressions were induced in the presence of VIT to balance the level of VIT for intestinal health. However, to elucidate the direct or indirect regulatory relationship between VIT accumulation and *pas-1*/*pas-3* expression, promoter activity assays would be valuable for understanding the molecular mechanisms underlying this interaction.

Although both *pas-1* and *pas-3* levels were increased by caffeine intake, their mode of action appeared to be different. The *pas-3* significantly repressed *unc-62* and decreased *vit* expression while *pas-1* seemed to regulate *unc-62* and *vit* expression at a low level under caffeine intake conditions. Further investigations, such as chromatin immunoprecipitation or transcription factor binding assays, will be necessary to determine whether there are any direct interactions between *pas* genes and *unc-62* under caffeine intake conditions. Moreover, we found that *pas-1*, but not *pas-3*, is required for the nuclear translocation of SKN-1 (the *C. elegans* ortholog of the mammalian Nrf protein) in response to caffeine intake. In *C. elegans*, SKN-1 is the master transcriptional regulator involving longevity, stress resistance, and lipid metabolism [48,49]. Its activation by nuclear translocation is essential for the regulation of lipid metabolism in response to nutrient status and oxidative stress during aging [49]. In this study, we suggest that caffeine intake induces SKN-1 activation through PAS-1. However, future studies are required to confirm and determine the interplay between *pas-1* and SKN-1 in regulating stress responses and longevity under caffeine intake conditions. Interestingly, it has been reported that *pas-3* may play a role in endocytosis, as *pas-3* RNAi resulted in an abnormal accumulation of VIT-2::GFP in the body [50]. We also observed that caffeine intake increased the expression of VIT-2::GFP in oocytes relative to the somatic tissues compared to caffeine-free conditions [10]. These findings suggest that the reduction in VIT in the body by caffeine intake is due to increased *pas-3* expression. However, the specific molecular pathways by which caffeine intake increases *pas-3* expression and subsequently enhances VIT transfer from the intestine to the oocyte remain unclear. Altogether, we suggest that *pas-1* mediates SKN-1 activation for lifespan extension but not *pas-3*. This distinction needs to be investigated further to understand the differential roles of these genes in proteasome function and lifespan regulation.

**Figure 5:**
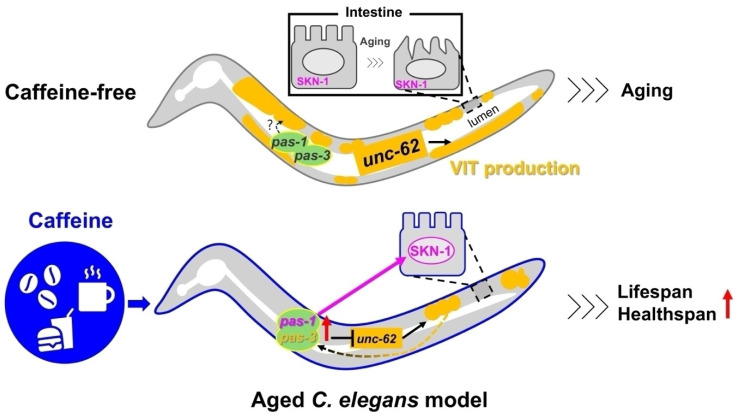
Working model. This model demonstrates that caffeine enhances intestinal health and extends lifespan in aged *C. elegans* through *pas-1* and *pas-3*. Caffeine intake increases the expression of these genes, resulting in a reduction in vitellogenin (VIT) levels by repressing *unc-62*. Furthermore, *pas-1* and *pas-3* are expressed in the presence of VIT, suggesting a possible feedback loop between VIT and proteasome activity that maintains VIT homeostasis for intestinal health. Caffeine also extends lifespan by activating SKN-1 via *pas-1*. Overall, caffeine promotes intestinal health and extends lifespan in an aged *C. elegans* model.

Many age-related diseases are characterized by protein misfolding and accumulation, often accompanied by decreased proteasome activity, leading to further protein accumulation with age [51,52]. Understanding the mechanisms underlying proteasome impairment in these diseases is needed to explore how proteasome activity can extend the healthspan. Our study revealed that the upregulation of proteasome genes *pas-1* and *pas-3* contributes to intestinal health and longevity by caffeine intake in aging *C. elegans*. These findings suggest that caffeine may provide insights into the mechanisms of proteasome impairment in age-related diseases, potentially supporting future therapeutic strategies. However, we still need to perform further analyses to clarify whether the effects we observed in this study are specific to caffeine by examining other controls such as caffeine analogs. In summary, we propose a working model describing the underlying mechanism of lifespan extended by caffeine intake (Figure 5).

## 5. Conclusions

This study provides substantial evidence showing the beneficial effects of caffeine on intestinal aging in aged *C. elegans* through enhanced proteasome activity, specifically involving *pas-1* and *pas-3*. Caffeine intake increased expressions of *pas-1* and *pas-3*, which reduced vitellogenin (VIT) production by repressing *unc-62*, a transcriptional activator of *vit* expression. Interestingly, *pas-1* and *pas-3* were expressed in the presence of *vit-2*, suggesting a feedback loop between VIT and proteasome activity to maintain the proper level of VIT for intestinal health. Furthermore, the lifespan extension by caffeine intake was mediated by the activation of SKN-1 through *pas-1*. However, the direct link between caffeine, *pas-1*, and SKN-1 activation remains speculative. In summary, caffeine intake maintains intestinal health through proteasome activity and extends lifespan in aged adults.

## Data Availability

The original contributions presented in this study are included in the article/Supplementary Materials. Further inquiries can be directed to the corresponding author.

## Supplementary Materials

The following supporting information can be downloaded at: https://www.mdpi.com/article/10.3390/nu16244298/s1, Figure S1: Embryonic lethality by *pas-1* and *pas-3* RNAi knockdown in *C. elegans*.; Table S1: qRT-PCR primers used in this study. Table S2: The list of genes which were differentially expressed in caffeine intake compared to caffeine-free.

## References

[1] Grubben M.J., Van Den Braak C.C., Broekhuizen R., De Jong R., Van Rijt L., De Ruijter E., Peters W.H., Katan M.B., Nagengast F.M. (2000). The Effect of Unfiltered Coffee on Potential Biomarkers for Colonic Cancer Risk in Healthy Volunteers: A Randomized Trial. Aliment. Pharmacol. Ther..

[2] Arnaud M.J. (1987). The Pharmacology of Caffeine. Prog. Drug Res..

[3] Reyes C.M., Cornelis M.C. (2018). Caffeine in the Diet: Country-Level Consumption and Guidelines. Nutrients.

[4] Saraiva S.M., Jacinto T.A., Gonçalves A.C., Gaspar D., Silva L.R. (2023). Overview of Caffeine Effects on Human Health and Emerging Delivery Strategies. Pharmaceuticals.

[5] Min H., Kawasaki I., Gong J., Shim Y.H. (2015). Caffeine Induces High Expression of *cyp-35A* Family Genes and Inhibits the Early Larval Development in *Caenorhabditis elegans*. Mol. Cells.

[6] Nawrot P., Jordan S., Eastwood J., Rotstein J., Hugenholtz A., Feeley M. (2003). Effects of Caffeine on Human Health. Food Addit. Contam..

[7] Gavrieli A., Yannakoulia M., Fragopoulou E., Margaritopoulos D., Chamberland J.P., Kaisari P., Kavouras S.A., Mantzoros C.S. (2011). Caffeinated Coffee Does Not Acutely Affect Energy Intake, Appetite, or Inflammation but Prevents Serum Cortisol Concentrations from Falling in Healthy Men. J. Nutr..

[8] Min H., Youn E., Shim Y.H. (2020). Maternal Caffeine Intake Disrupts Eggshell Integrity and Retards Larval Development by Reducing Yolk Production in a *Caenorhabditis elegans* Model. Nutrients.

[9] Takahashi K., Yanai S., Shimokado K., Ishigami A. (2017). Coffee Consumption in Aged Mice Increases Energy Production and Decreases Hepatic MTOR Levels. Nutrition.

[10] Min H., Youn E., Shim Y.H. (2021). Long-Term Caffeine Intake Exerts Protective Effects on Intestinal Aging by Regulating Vitellogenesis and Mitochondrial Function in an Aged *Caenorhabditis elegans* Model. Nutrients.

[11] Min H., Lee M., Kang S., Shim Y.H. (2024). Vitamin B12 Supplementation Improves Oocyte Development by Modulating Mitochondria and Yolk Protein in a Caffeine-Ingested *Caenorhabditis elegans* Model. Antioxidants.

[12] Sheng X., Zhu Y., Zhou J., Yan L., Du G., Liu Z., Chen H. (2021). Antioxidant Effects of Caffeic Acid Lead to Protection of *Drosophila* Intestinal Stem Cell Aging. Front. Cell Dev. Biol..

[13] González S., Salazar N., Ruiz-Saavedra S., Gómez-Martín M., de los Reyes-Gavilán C.G., Gueimonde M. (2020). Long-Term Coffee Consumption Is Associated with Fecal Microbial Composition in Humans. Nutrients.

[14] Baspinar B., Eskici G., Ozcelik A.O. (2017). How Coffee Affects Metabolic Syndrome and Its Components. Food Funct..

[15] O’Keefe J.H., DiNicolantonio J.J., Lavie C.J. (2018). Coffee for Cardioprotection and Longevity. Prog. Cardiovasc. Dis..

[16] Gkegkes I.D., Minis E.E., Iavazzo C. (2020). Effect of Caffeine Intake on Postoperative Ileus: A Systematic Review and Meta-Analysis. Dig. Surg..

[17] Singh R.K., Chang H.W., Yan D., Lee K.M., Ucmak D., Wong K., Abrouk M., Farahnik B., Nakamura M., Zhu T.H. (2017). Influence of Diet on the Gut Microbiome and Implications for Human Health. J. Transl. Med..

[18] Li Z., Zhang Z., Ren Y., Wang Y., Fang J., Yue H., Ma S., Guan F. (2021). Aging and Age-related Diseases: From Mechanisms to Therapeutic Strategies. Biogerontology.

[19] Lee K.X., Quek K.F., Ramadas A. (2023). Dietary and Lifestyle Risk Factors of Obesity Among Young Adults: A Scoping Review of Observational Studies. Curr. Nutr. Rep..

[20] Zhang K., Ma Y., Luo Y., Song Y., Xiong G., Ma Y., Sun X., Kan C. (2023). Metabolic Diseases and Healthy Aging: Identifying Environmental and Behavioral Risk Factors and Promoting Public Health. Front. Public Health.

[21] Surugiu R., Iancu M.A., Vintilescu Ș.B., Stepan M.D., Burdusel D., Genunche-Dumitrescu A.V., Dogaru C.A., Dumitra G.G. (2024). Molecular Mechanisms of Healthy Aging: The Role of Caloric Restriction, Intermittent Fasting, Mediterranean Diet, and Ketogenic Diet—A Scoping Review. Nutrients.

[22] Drozdowski L., Thomson A.B. (2006). Aging and the Intestine. World J. Gastroenterol..

[23] McGee M.D., Weber D., Day N., Vitelli C., Crippen D., Herndon L.A., Hall D.H., Melov S. (2011). Loss of Intestinal Nuclei and Intestinal Integrity in Aging *C. elegans*. Aging Cell.

[24] Perez M.F., Lehner B. (2019). Vitellogenins—Yolk Gene Function and Regulation in *Caenorhabditis elegans*. Front. Physiol..

[25] Baker M.E. (1988). Is Vitellogenin an Ancestor of Apolipoprotein B-100 of Human Low-Density Lipoprotein and Human Lipoprotein Lipase?. Biochem. J..

[26] Kimbleand J., Sharrock W.J. (1983). Tissue-Specific Synthesis of Yolk Proteins in *Caenorhabditis elegans*. Dev. Biol..

[27] Ezcurra M., Benedetto A., Sornda T., Gilliat A.F., Au C., Zhang Q., van Schelt S., Petrache A.L., Wang H., de la Guardia Y. (2018). *C. elegans* Eats Its Own Intestine to Make Yolk Leading to Multiple Senescent Pathologies. Curr. Biol..

[28] Sornda T., Ezcurra M., Kern C., Galimov E.R., Au C., De La Guardia Y., Gems D. (2019). Production of YP170 Vitellogenins Promotes Intestinal Senescence in *Caenorhabditis elegans*. J. Gerontol. A Biol. Sci. Med. Sci..

[29] Song R., Hu M., Qin X., Qiu L., Wang P., Zhang X., Liu R., Wang X. (2023). The Roles of Lipid Metabolism in the Pathogenesis of Chronic Diseases in the Elderly. Nutrients.

[30] Libina N., Berman J.R., Kenyon C. (2003). Tissue-Specific Activities of *C. elegans* DAF-16 in the Regulation of Lifespan. Cell.

[31] Brenner S. (1974). The genetics of *Caenorhabditis elegans*. Genetics.

[32] Hoogewijs D., Houthoofd K., Matthijssens F., Vandesompele J., Vanfleteren J.R. (2008). Selection and Validation of a Set of Reliable Reference Genes for Quantitative Sod Gene Expression Analysis in *C. elegans*. BMC Mol. Biol..

[33] Maeda I., Kohara Y., Yamamoto M., Sugimoto A. (2001). Large-Scale Analysis of Gene Function in *Caenorhabditis elegans* by High-Throughput RNAi. Curr. Biol..

[34] Takahashi M., Iwasaki H., Inoue H., Takahashi K. (2002). Reverse Genetic Analysis of the *Caenorhabditis elegans* 26S Proteasome Subunits by RNA Interference. Biol. Chem..

[35] Walker A.C., Bhargava R., Vaziriyan-Sani A.S., Pourciau C., Donahue E.T., Dove A.S., Gebhardt M.J., Ellward G.L., Romeo T., Czyż D.M. (2021). Colonization of the *Caenorhabditis elegans* gut with human enteric bacterial pathogens leads to proteostasis disruption that is rescued by butyrate. PLoS Pathog..

[36] Davy A., Bello P., Thierry-Mieg N., Vaglio P., Hitti J., Doucette-Stamm L., Thierry-Mieg D., Reboul J., Boulton S., Walhout A.J. (2001). A Protein-Protein Interaction Map of the *Caenorhabditis elegans* 26S Proteasome. EMBO Rep..

[37] Papaevgeniou N., Chondrogianni N. (2014). The Ubiquitin Proteasome System in *Caenorhabditis elegans* and Its Regulation. Redox Biol..

[38] Vilchez D., Saez I., Dillin A. (2014). The Role of Protein Clearance Mechanisms in Organismal Ageing and Age-Related Diseases. Nat. Commun..

[39] Kaushik S., Cuervo A.M. (2015). Proteostasis and Aging. Nat. Med..

[40] Dowen R.H. (2019). CEH-60/PBX and UNC-62/MEIS Coordinate a Metabolic Switch That Supports Reproduction in *C. elegans*. Dev. Cell.

[41] Macqueen A.J., Baggett J.J., Perumov N., Bauer R.A., Januszewski T., Schriefer L., Waddle J.A. (2005). ACT-5 Is an Essential *Caenorhabditis elegans* Actin Required for Intestinal Microvilli Formation. Mol. Biol. Cell.

[42] Anderson R.T., Bradley T.A., Smith D.M. (2022). Hyperactivation of the Proteasome in *Caenorhabditis elegans* Protects against Proteotoxic Stress and Extends Lifespan. J. Biol. Chem..

[43] Tavirani M.R., Farahani M., Tavirani M.R., Razzaghi Z., Arjmand B., Khodadoost M. (2024). Introducing Coffee as a Complementary Agent Beside Metformin Against Type 2 Diabetes. Res. J. Pharmacogn..

[44] Lim H.H., Kim O.Y. (2016). Association of Serum Apolipoprotein B with the Increased Risk of Diabetes in Korean Men. Clin. Nutr. Res..

[45] Hashmi S., Wang Y., Parhar R.S., Collison K.S., Conca W., Al-Mohanna F. (2013). A *C. elegans* Model to Study Human Metabolic Regulation. Nutr. Metab..

[46] Mikkonen E., Haglund C., Holmberg C.I. (2017). Immunohistochemical Analysis Reveals Variations in Proteasome Tissue Expression in *C. elegans*. PLoS ONE.

[47] Li X., Matilainen O., Jin C., Glover-Cutter K.M., Holmberg C.I., Blackwell T.K. (2011). Specific SKN-1/NrF Stress Responses to Perturbations in Translation Elongation and Proteasome Activity. PLoS Genet..

[48] Lynn D.A., Dalton H.M., Sowa J.N., Wang M.C., Soukas A.A., Curran S.P., Ruvkun G. (2015). Omega-3 and -6 Fatty Acids Allocate Somatic and Germline Lipids to Ensure Fitness during Nutrient and Oxidative Stress in *Caenorhabditis elegans*. Proc. Natl. Acad. Sci. USA.

[49] Blackwell T.K., Steinbaugh M.J., Hourihan J.M., Ewald C.Y., Isik M. (2015). SKN-1/Nrf, Stress Responses, and Aging in *Caenorhabditis elegans*. Free Radic. Biol. Med..

[50] Balklava Z., Pant S., Fares H., Grant B.D. (2007). Genome-Wide Analysis Identifies a General Requirement for Polarity Proteins in Endocytic Traffic. Nat. Cell Biol..

[51] Saez I., Vilchez D. (2014). The Mechanistic Links Between Proteasome Activity, Aging and Age-Related Diseases. Curr. Genom..

[52] Tomaru U., Takahashi S., Ishizu A., Miyatake Y., Gohda A., Suzuki S., Ono A., Ohara J., Baba T., Murata S. (2012). Decreased Proteasomal Activity Causes Age-Related Phenotypes and Promotes the Development of Metabolic Abnormalities. Am. J. Pathol..

