# Supplementary material for: Caffeine-Induced Upregulation of pas-1 and pas-3 Enhances Intestinal Integrity by Reducing Vitellogenin in Aged Caenorhabditis elegans Model
Source: Nutrients. 2024 Dec 12;16(24):4298. doi: 10.3390/nu16244298 (PMC11677849; doi:10.3390/nu16244298)
Supplement: Supplementary file 1 [file nutrients-16-04298-s001.zip › Supplementary table S1.pdf]

Table S1. qRT-PCR primers used in this study.

mRNA Target		Primer Sequence	GenBank Database
act-1	forward	5′-CCAGGAATTGCTGATCGTATGCAGAA-3′	NC_003283.11, Chr V:
	reverse	5′-TGGAGAGGGAAGCGAGGATAG-3′	11081052.11082415: 133 bp
pas-1	forward	5′-TCGCTGATACCGTCACTTCG-3′	NC_003283.11, Chr V:
	reverse	5′-AGCACATCCCAAACTACGCA-3′	14421566.14422618, complement:
pas-2	forward	5′-TTCGCATGGAAAGCAACAGC-3′	NC_003283.11, Chr V:
	reverse	5′-TTGGTGAGCCGATGGAATCC-3′	10770368.10771351:
pas-3	forward	5′-TTCGGACAACATCTCGTGCA-3′	NC_003279.8m Chr I:
	reverse	5′-GAAGTGACACTCCGAACGGT-3′	5105110.5106048, complement:
pas-4	forward	5′-CGTCGCCCATTCGGAATTTC-3′	NC_003279.8 Chr I:
	reverse	5′-ACCTGAGCCAGCGACTTTAC-3′	8727218.8728487, complement:
pas-5	forward	5′-ATGGTCAATGACGCGATCGA-3′	NC_003279.8 Chr I:
	reverse	5′-CGTCGTCGTCTCCAAACTGA-3′	10566350.10567300:
pas-6	forward	5′-CAAGGTTCGGCAACTGTTGG-3′	NC_003283.11 Chr V:
	reverse	5′-ACCGCTTGCTTGTAATCCCA-3′	5583161.5584343, complement:
pas-7	forward	5′-GTTTGGAGCAGGAGCCTTCT-3′	NC_003280.10 Chr II:
	reverse	5′-GAGCAGCTTCCTTCACGAGT-3′	10098782.10099854, complement:
vit-2	forward	5′-CTCAAGAACGAGGAGTGCGAA-3′	NC_003284.9, Chr X:
	reverse	5′-AAGTGCCGGTCTAGCTTAA-3′	5101042.5106169:
vit-6	forward	5′-ACCCCATGCTACTCCGTTCTC-3′	NC_003282.8, Chr IV:
	reverse	5′-GATGGGAGGCAGTAGACGGAG-3′	8261157.8266459:
unc-62	forward	5′-TAAGACATACCCAAGAGAATGCTG-3′	NC_003283.11, Chr V:
	reverse	5′-TTTGCCTTTCAGACAGACCA-3′	4497463.4511447:
