# Supplementary figures and images for: Caffeine-Induced Upregulation of pas-1 and pas-3 Enhances Intestinal Integrity by Reducing Vitellogenin in Aged Caenorhabditis elegans Model
Source: Nutrients. 2024 Dec 12;16(24):4298. doi: 10.3390/nu16244298 (PMC11677849; doi:10.3390/nu16244298)

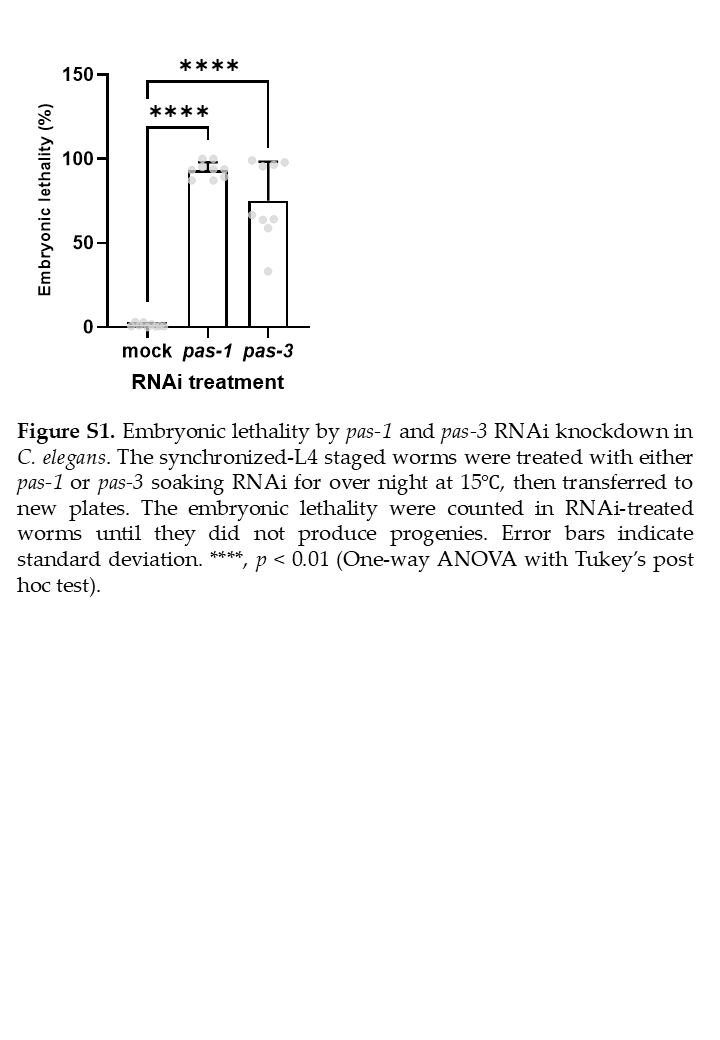

Supplement: Supplementary file 1 [file nutrients-16-04298-s001.zip › Figure S1.tif]
